# Comparison of Microtox and Xenoassay Light as a Near Real Time River Monitoring Assay for Heavy Metals

**DOI:** 10.1155/2014/834202

**Published:** 2014-04-01

**Authors:** M. I. E. Halmi, Hussain Jirangon, W. L. W. Johari, A. R. Abdul Rachman, M. Y. Shukor, M. A. Syed

**Affiliations:** ^1^Department of Biochemistry, Faculty of Biotechnology and Biomolecular Sciences, Universiti Putra Malaysia, UPM Serdang, 43400 Selangor, Malaysia; ^2^Department of Environmental Science, Faculty of Environmental Studies, Universiti Putra Malaysia, UPM Serdang, 43400 Selangor, Malaysia; ^3^Centre of Excellence for Environmental Forensics, Universiti Putra Malaysia, UPM Serdang, 43400 Selangor, Malaysia

## Abstract

Luminescence-based assays for toxicants such as Microtox, ToxAlert, and Biotox have been used extensively worldwide. However, the use of these assays in near real time conditions is limited due to nonoptimal assay temperature for the tropical climate. An isolate that exhibits a high luminescence activity in a broad range of temperatures was successfully isolated from the mackerel, *Rastrelliger kanagurta*. This isolate was tentatively identified as *Photobacterium* sp. strain MIE, based on partial 16S rDNA molecular phylogeny. Optimum conditions that support high bioluminescence activity occurred between 24 and 30°C, with pH 5.5 to 7.5, 10 to 20 g/L of sodium chloride, 30 to 50 g/L of tryptone, and 4 g/L of glycerol as the carbon source. Assessment of near real time capability of this bacterial system, Xenoassay light to monitor heavy metals from a contaminated river running through the Juru River Basin shows near real time capability with assaying time of less than 30 minutes per samples. Samples returned to the lab were tested with a standard Microtox assay using *Vibrio fishceri*. Similar results were obtained to Xenoassay light that show temporal variation of copper concentration. Thus, this strain is suitable for near real time river monitoring of toxicants especially in the tropics.

## 1. Introduction

The extensive industrial and agricultural activities in Malaysia have resulted in the increase of pollutants such as heavy metals, pesticides, and organic and inorganic solvents in the environment. Malaysian Department of Environment (DOE) in 2011 has categorized 189 out of 464 rivers as polluted or slightly polluted [1], which is an issue of concern due to the toxicity, carcinogenicity, and mutagenicity of the pollutants. Toxicants can cause potentially harmful effects to human beings, aquatic organisms, and food webs because some of them cannot be fully degraded [2]. Toxicants, especially heavy metals, are toxic to aquatic organisms. Therefore, it is important to periodically monitor toxicants in the environment [3]. A simple and fast procedure is required to screen for the presence of toxic substances from industrial effluents, polluted rivers, and other polluted locations [4, 5].

Bioluminescence-based systems are suitable for preliminary screening of toxicants in the environment. This system is sensitive to many toxicants and utilizes a rapid and simple operation [6]. Despite its simplicity, it can represent the real impact of all chemicals present in a given sample or ecosystems [7]. The system involves bioluminescent bacteria, which are widely distributed in marine, freshwater, and terrestrial environment [8]. Bioluminescence is an aerobic oxidation process and the enzyme involved in the production of luminescence is luciferase. The enzyme catalyzes the oxidation of its substrate, luciferin, and is mediated by a reduced coenzyme, flavin mononucleotide. The interactions of toxicants with the bioluminescent bacteria cause the inhibition of the luminescence production [7]. The effect of toxicants on the microorganisms can be determined within 30 minutes or less depending on the types of toxicants [9]. An example of a commercially produced bioassay using bioluminescent bacterial cultures is the Microtox system [10]. This system uses the bacterium* Vibrio fischeri *and has an optimal assay temperature of 15°C [11, 12]. Deviations of a few degrees Celsius from this temperature can dramatically affect the luminescence production [13]. The system requires a refrigerated water bath, which is not practical, expensive to maintain, and instrument-dependent for field applications in the tropical regions [14]. A tropical country such as Malaysia can exhibit a wide variation of daily temperatures ranging from 23 at night to 34°C in the afternoon [15]. Other commercial bioluminescence-based systems such as ToxAlert also require an exact assay temperature at 15°C and Biotox as well as Toxi-Screening Kit is stable between 15 and 25°C [13]. Thus, a tropical bioluminescent bacterium that could cover this broad tropical temperature range is urgently needed.

In this work, we have isolated* Photobacterium *sp. strain MIE from mackerel (*Rastrelliger kanagurta*) from the local market. The tropical isolate is capable of high luminescent activity within a broad range of temperature from 24°C to 30°C. This study aims to characterize the optimum conditions for growth and the environmental factors that are essential for bioluminescence production. A field trial has been conducted on Malaysian water to demonstrate the near real time application of this bacterium.

## 2. Materials and Methods

### 2.1. Chemicals

All chemicals used were of analytical grade and purchased either from Sigma (St. Louis, MO, USA), Fermentas (USA), Merck (Darmstadt, Germany), or Qiagen (Valencia, CA, USA).

### 2.2. Isolation and Screening of Bioluminescent Bacteria

The bioluminescent bacteria were screened from different marine organisms and sea waters. Marine organisms such as sardines, mackerel, squids, and prawn were bought from the Sri Serdang Market, Selangor, in November 2010. Seawater was collected from Port Dickson, Negeri Sembilan, and Kuala Juru, Pulai Pinang, in December 2010. The marine organisms were half-submerged in 3% (w/v) of NaCl solution for 24 hours at 20°C. After 24 hours of incubation, 1 mL from each sample was diluted 10 times and 50 *μ*L was spread on luminescence agar containing the following (per L): 10 g NaCl, 10 g peptone, 3 mL glycerol, 3 g yeast extract, and 18 g agar. The plates were incubated at room temperature (27°C) for 24 hours until the bacterial colonies were visible [9, 16, 17]. For screening purpose, a single colony was inoculated into 200 mL conical flask containing 50 mL of the luminescent broth and incubated at 100 rpm on a rotary shaker for 12 hours at room temperature.

### 2.3. Identification of Bacterium

Identification of strain MIE was done by using several tests mainly on the basis of its cultural and morphological characteristics (micro- and macromorphological features) on the nutrient agar, gram staining, and followed by sequence analysis of 16S ribosomal RNA. 16S rRNA analysis provides a powerful phylogenetic framework for classification of organisms [18]. Genomic DNA extraction was performed by alkaline lysis using DNeasy Blood & Tissue Purification Kit (Qiagen, USA). 16S rRNA genes were amplified by using genomic DNA and universal primer pair (5′-AGAGTTTGATCATGGCTGAG-3′ and 5′-ACGGTTACCTTGTTACGACTT-3′). The amplicons were purified prior to sequencing. The resultant bases were compared with the GenBank database using the Blast server at NCBI (http://www.ncbi.nlm.nih.gov/BLAST/). The 16s rRNA ribosomal gene sequence was deposited in GenBank under Accession number* JX020946*.

### 2.4. Phylogenetic Analysis

A multiple alignments of 19 different 16S rRNA gene sequences, which closely match strain MIE, were retrieved from GenBank and aligned using clustal W [19] with the PHYLIP output option. The alignment was checked by eye for any obvious misalignments. Alignment positions with gaps were excluded from the calculations. A phylogenetic tree was constructed by using PHYLIP, version 3.573 (J. Q. Felsenstein, PHYLIP—phylogeny inference package, version 3.573, (http://evolution.genetics.washington.edu/phylip.html)), with* Rhodococcus* sp. YB1 as the outgroup in the cladogram. The four models of nucleotide substitution are those of [20–22], the F84 model [23], and the model underlying the Log Det distance [24]. Phylogenetic tree was inferred by using the neighbor-joining method [25]. With each algorithm, confidence levels for individual branches within the tree were checked by repeating the PHYLIP analysis with 1000 bootstraps [26] by the SEQBOOT program in the PHYLIP package. Majority rule (50%) consensus trees were constructed for the topologies found using a family of consensus tree methods called the Ml methods using the CONSENSE program and the tree was viewed using Tree View [27].

### 2.5. Characterization of Bioluminescence Production of Isolated Bioluminescent Bacterium

It is important to investigate the best condition for the selected isolate to produce an optimum and stable luminescence [28]. The bacterial cultures were tested on different types of carbon and nitrogen sources, in addition to wide ranges of NaCl concentrations, pHs, and temperatures.

### 2.6. Measurement of Luminescence

Luminescence was measured using a Beckman Counter DTX 800 multimode detector and reported as Relative Luminescence Unit (RLU). 200 *μ*L of samples was collected in DTX microplate 96 wells before the readings were taken. The measurements were taken in triplicate.

### 2.7. Identification of Responsible Luciferase Gene by Specific PCR

The total genomic DNA of* Photobacterium* strain MIE was extracted by using Thermo Genomic kit and used as template for polymerase chain reaction (PCR) for detection of the presence luciferase gene in* Photobacterium* strain MIE genomic. PCR assay was performed in 12.5 *μ*L reaction mixture, containing 1 *μ*L genomic DNA solution, 2 U of* Taq* polymerase (Bioline, London, U.K), 1X MyFi reaction buffer including dNTP's, MgCl_2_ and enhancer (Bioline, London, U.K), and 20 pmol for each specific primer (Firstbase, Malaysia).

Amplification reaction was performed by using Biorad thermocycler (Biorad). First step is initial denaturation that was applied for 1 min at 95°C. After that, 30 cycles were performed, consisting of 15 s denaturation at 95°C, 15 s annealing at 55°C, and 90 s extension at 72°C, followed by 7 min final extension at 72°C and cooling at 10°C. The PCR mixture was viewed using 1% agarose gel electrophoresis by using 1 kb DNA ladder mixture marker (Fermentas).

### 2.8. Near Real-Time Biomonitoring Field Trials

The bioluminescent bacterium* Photobacterium *sp. strain MIE was cultured in luminescence broth for 12 hours at room temperature. After a 12-hour incubation, the bacterial cell was harvested by centrifugation (10000 ×g, 10 min) and the pellet was diluted using minimal salt media (MSM) (1 L of distilled water was supplemented with 12.8 g Na_2_HPO_4_·7H_2_O, 3.1 g KH_2_PO_4_, 17 g NaCl, 1 g NH_4_Cl, 0.5 g MgSO_4_, and 3 mL glycerol), which has been used as substitute media for toxicity assay [29]. High density of bacterial culture can also affect the sensitivity and luminescence of the bioassay. The culture was further diluted 1000 times so that the emission was within the range of 50,000 to 100,000 RLU prior to assaying [9]. The assay was conducted by mixing 20 *μ*L of water samples with 180 *μ*L of bacterial cultures. One location was selected and used to demonstrate near real time applicability of the developed assay. Water samples were obtained every two hours for a period of one day from a large drain at the Prai Industrial Park (N 05°20.87′ E 100°24.692′). This area was chosen as it was previously reported with high concentrations of heavy metals [4, 30]. The total time per assay was approximately 30 minutes and luminescence was measured with a portable luminometer (MicroBioTests Inc., Mariakerke (Gent, Belgium)).

### 2.9. Microtox Toxicity Assay

The assay method of Microtox was carried out by following the recommended procedure as suggested by the manufacturer with minor modification. Briefly, 1 mL of chilled distilled water was added into a vial of freeze-*dried Photobacterium phosphoreum* cells (Arachem Sdn. Bhd) and slowly mixed by swirling the vial to reconstitute the cells. The cells must be used within a few hours. Quality control of the assay performance can be validated using a phenol standard solution (10 mg/L). Appropriately diluted samples were prepared in 2% sodium chloride solution and about 10^6^ cells were added to the dilution vials. An emission of between the range of 20,000 and 40,000 RLU was obtained. Measurement of luminescence (Beckman Counter DTX 800 multimode detector) at time zero and after 30 min was carried out and compared to control solution (sodium chloride 2%) minus the tested compound. All the assay and dilution operations were carried out at 15 ± 0.5°C [31].

### 2.10. Determination of Heavy Metals

The determination of heavy metals in the samples was carried out using atomic emission spectrometry on ICP-OES (Optima 3700DV, Perkin-Elmer, USA). All experiments were performed in triplicate.

### 2.11. Statistical Analysis

All data were analyzed using Graphpad Prism version 5.0. Values are means ± standard errors. The comparison between groups was performed using a Student's *t*-test or a one-way analysis of variance with post-hoc analysis by Tukey's test. *P* < 0.05 was considered statistically significant.

## 3. Results

### 3.1. Isolation, Screening, and Identification of Bioluminescent Bacteria

**Figure 1:**
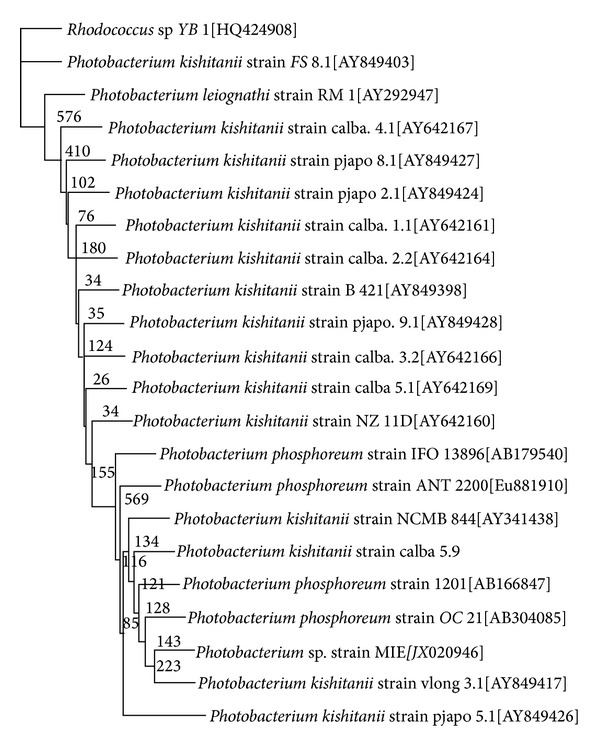
Phylogram (neighbour-joining method) showing the genetic relationship between* Photobacterium* sp. strain MIE and other related references microorganisms based on the 16S rRNA gene sequence analysis from the GenBank database.* Rhodococcus *sp. is the outgroup. Species names were followed by the accession numbers of 16S rRNA. The internal labels at the branching points refer to bootstrap value. Scale bar represents 100 nucleotides substitution.

In this study, isolates were chosen based on the ability to produce strong luminescence at room temperature (27°C). Among the 15 luminescent isolates, only isolate K10 (isolated from mackerel) was able to grow in the luminescence media with good intensity of blue-green light after 12 hours of incubation period at room temperature (data not shown). Isolate K10 was found to be a gram-negative and rod-shaped bacterium. Through 16S rRNA sequence analysis, a low bootstrap value (22.3%) links isolate K10 to* P. kishitanii* strain vlong 1.3, indicating a low phylogenetic relationship (Figure 1). This bacterium is grouped with* Photobacterium* species, albeit in a separate branch, which conversely have low bootstrap values (<50%), implying that its phylogenetic position might still be modified. This bacterium is assigned tentatively as* Photobacterium* sp. strain MIE with Accession number of JX020946.

### 3.2. Characterization of Bioluminescence Production

#### 3.2.1. Effect of Carbon Sources

**Figure 2:**
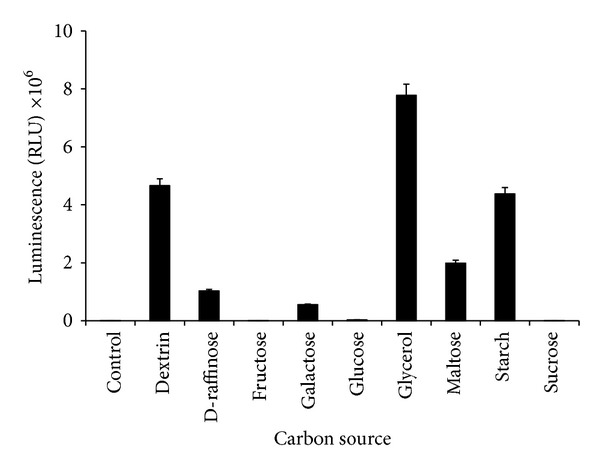
Effects of different carbon sources on bioluminescence production of* Photobacterium* sp. strain MIE. The isolate was grown in luminescence media containing 1 g/L of carbon sources incubated in orbital shaker (100 rpm) for 12 hours at room temperature. Data is mean ± standard error of the mean (*n* = 3). The cultures were grown without any carbon source as control.

**Figure 3:**
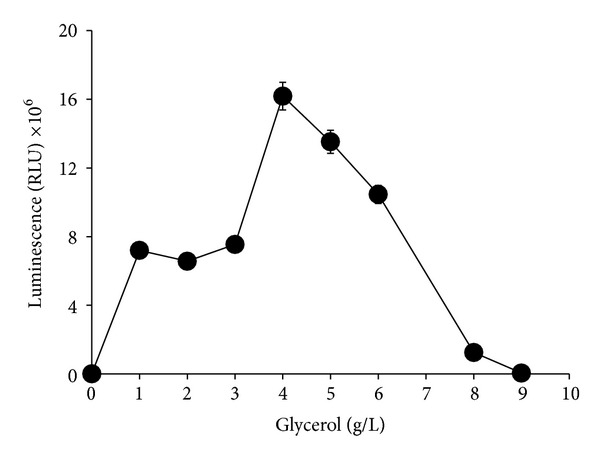
Effects of glycerol concentration on bioluminescence production of* Photobacterium* sp. strain MIE. The cultures were grown in luminescence media containing different concentration of glycerol incubated in orbital shaker (100 rpm) for 12 hours at room temperature. Data is mean ± standard error of the mean (*n* = 3).

Carbon source was an important luminescence parameter to this bacterium as luminescence activity was not observed in the medium without carbon source (Figure 2). Out of the nine carbon sources tested, only glycerol produced the highest significant (*P* < 0.05) luminescence while there was no significant difference in terms of bioluminescence production for starch and dextrin (*P* > 0.05). Other carbon sources such as D-raffinose, galactose, maltose, and glucose showed bioluminescence production but at much lower levels. The effect of different concentrations of glycerol on luminescence production showed that the optimum concentration of glycerol was 4 g/L (*P* < 0.05) after 12 hours of incubation period (Figure 3).

#### 3.2.2. Effect of Nitrogen Source

**Figure 4:**
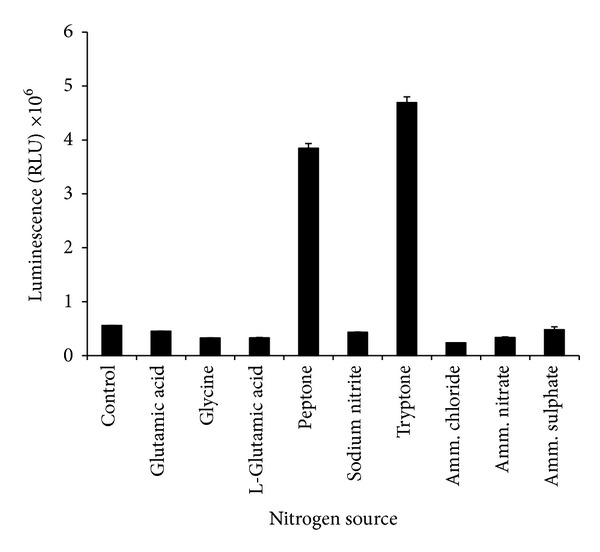
Effects of different nitrogen sources on bioluminescence production by* Photobacterium* sp. strain MIE. The cultures were grown in luminescence media containing 10 g/L nitrogen sources and incubated in an orbital shaker (100 rpm) for 12 hours at room temperature. Data is mean ± standard error of the mean (*n* = 3). The cultures were grown without nitrogen source as control.

**Figure 5:**
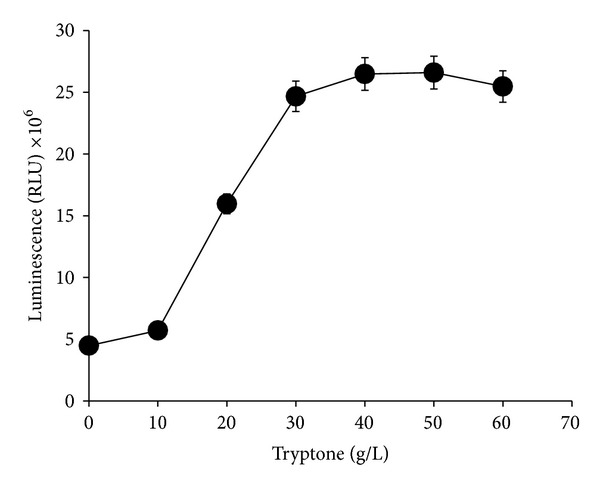
Effects of different tryptone concentrations on bioluminescence production of* Photobacterium* sp. strain MIE. The cultures were grown in luminescence media containing different concentrations of tryptone and incubated in an orbital shaker (100 rpm) for 12 hours at room temperature. Data is mean ± standard error of the mean (*n* = 3).

High emission of luminescence was observed when the culture was grown with either peptone or tryptone (Figure 4) but tryptone gave a significantly higher (*P* < 0.05) luminescence than peptone. Other nitrogen sources tested did not support luminescence activity in this bacterium. The optimum concentration of tryptone was between 30 and 40 g/L (Figure 5).

#### 3.2.3. Effect of Salinity

**Figure 6:**
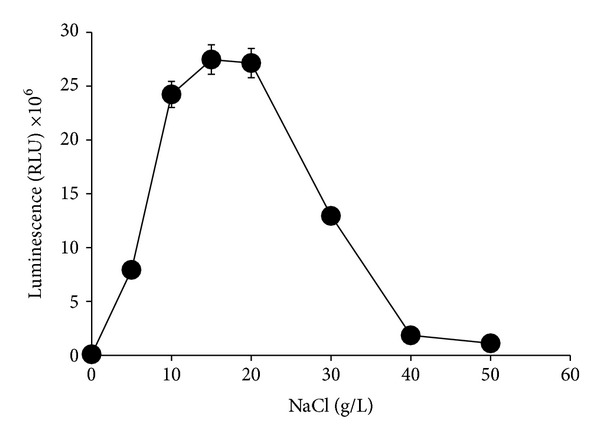
Effects of sodium chloride on bioluminescence production by* Photobacterium* sp. strain MIE. The cultures were grown in luminescence media containing different concentrations of sodium chloride and incubated in an orbital shaker (100 rpm) for 12 hours at room temperature. Data is mean ± standard error of the mean (*n* = 3).

The concentration of sodium chloride required to produce high luminescence activity was between 10 and 20 g/L with no significant difference (*P* > 0.05) between the values obtained after 12 hours incubation time in luminescence media. Luminescence activities dramatically dropped below and above this range (Figure 6).

#### 3.2.4. Effect of pH

**Figure 7:**
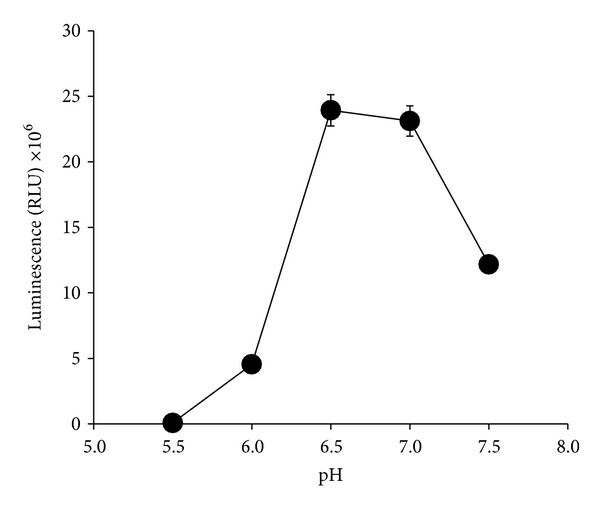
Effects of different pHs on bioluminescence production by* Photobacterium* sp. strain MIE in luminescence media. The isolate was grown in luminescence media at different pH and incubated in an orbital shaker (100 rpm) for 12 hours at room temperature. Data is mean ± standard error of the mean (*n* = 3).

This bacterium was able to produce high luminescence at pH between 5.5 and 7.5 with no significant difference (*P* > 0.05) between the pH values after 12 hours incubation time in luminescence media. Luminescence production of the bacterium ceased completely outside of this range (Figure 7).

#### 3.2.5. Effect of Temperature

**Figure 8:**
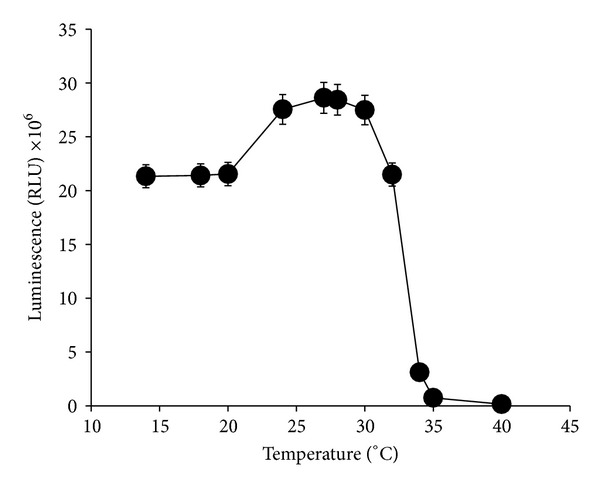
Effects of different temperatures on bioluminescence produced by* Photobacterium* sp. strain MIE. The cultures were grown in luminescence media at different temperatures and incubated in an orbital shaker (100 rpm) for 12 hours. Data is mean ± standard error of the mean (*n* = 3).

Stable production of bioluminescence was observed between 24 and 30°C with no significant differences (*P* > 0.05) between the temperatures. At temperatures lower than this range especially between 15 and 20°C, luminescence fell to about 75% of optimal value while at temperatures higher than the optimal values, luminescence ceased completely (Figure 8).

#### 3.2.6. Profile of Bioluminescence Production and Bacterial Growth

**Figure 9:**
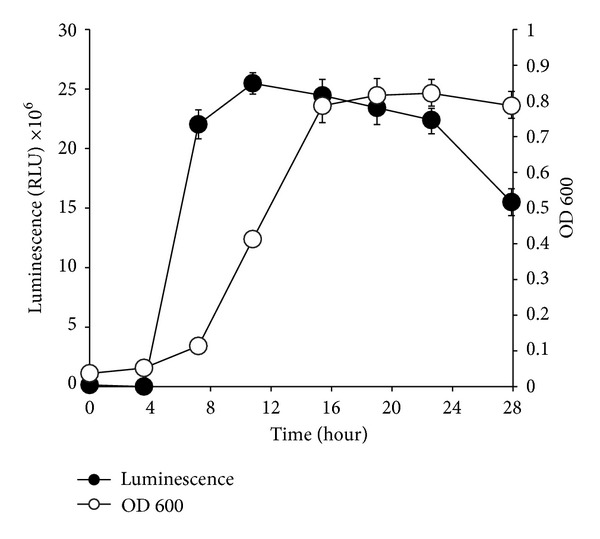
Bioluminescence production and bacterial growth of* Photobacterium* sp. strain MIE in luminescence media over time. The isolate was grown in luminescence media and incubated in an orbital shaker (100 rpm) for 28 hours at room temperature. Bioluminescence production (black circle) and bacterial growth (circle) were determined using Beckman Counter DTX 800 multimode detector. The error bars represent the mean ± standard deviation of three replicates.

**Figure 10:**
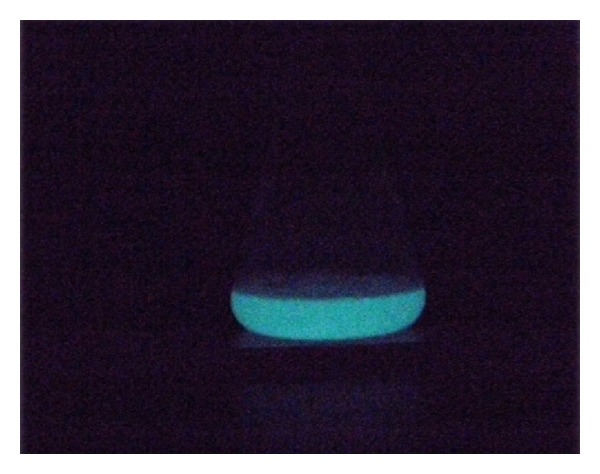
The bacterial cultures produced luminescence in dark condition when grown in luminescence broth for 12 hours at room temperature (27°C).

In order to determine the overall maximum bioluminescence production,* Photobacterium* sp. strain MIE was grown under the optimal conditions as described in the previous sections. The growth and luminescence curves of this bacterium are presented in Figure 9. The absorbance value was correlated with the bacterial number which represents bacterial growth. The luminescence was correlated with bacterial number and luminescent efficiency. The results show that the luminescence peak corresponds to bacterial growth and the luminescence production started to increase during the logarithmic growth phase. Luminescence only appeared after 8 hours of incubation. The bacterial cultures produced peak luminescence between 12 and 24 hours of incubation and started to decline above 24 hours (Figure 9). The bioluminescence of* Photobacterium* sp. strain MIE grown in shake flask taken in the dark is shown in Figure 10.

### 3.3. Isolation and Sequencing of Luciferase Gene

**Figure 11:**
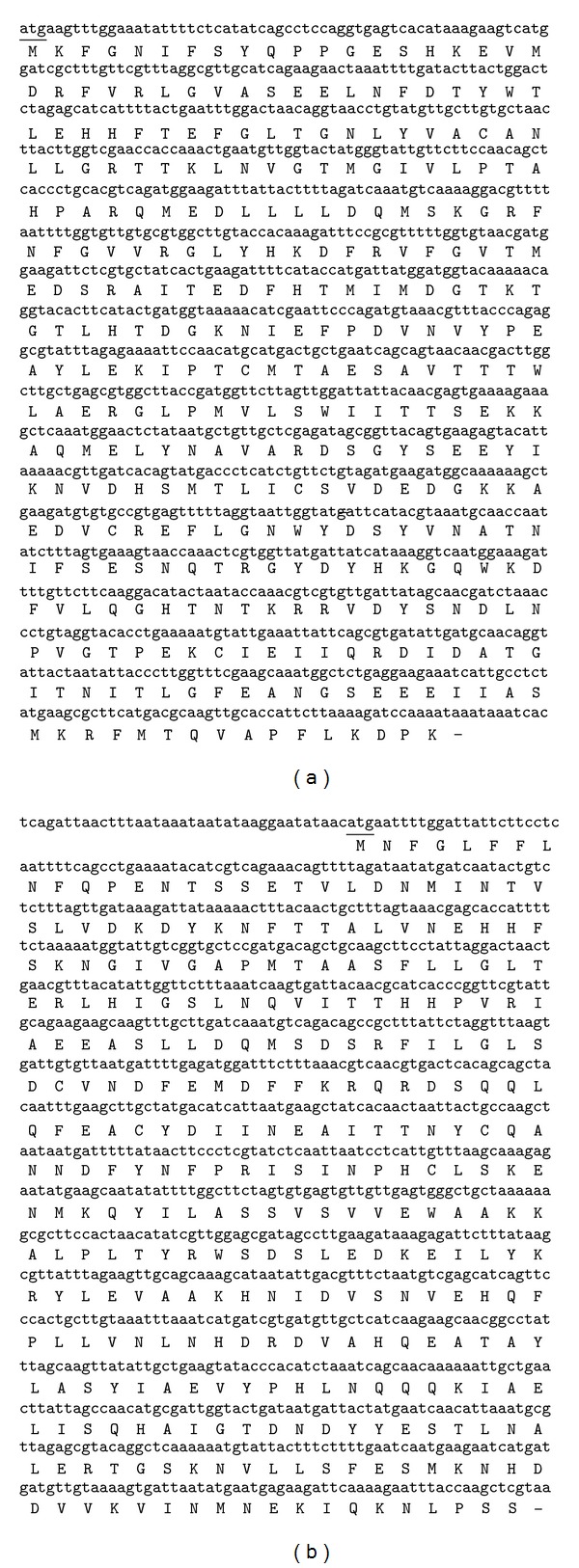
Full length nucleotide and amino acid sequences of luciferase. (a) and (b) sequences indicate nucleotide and amino acid sequence, respectively. The first nucleotide and amino acid of lux A and lux B of* Photobacterium* sp. strain MIE are underlined.

Luciferase genes were successfully amplified using luciferase-specific primers. The expected size of luciferase nucleotide sequence was approximately 2.1 kbp. New isolated luciferase genes from* Photobacterium* strain MIE consisted of alpha and beta subunits. Based on Figure 11, alpha and beta subunits of the luciferase gene contained 1071 nucleotide sequences encoding 356 amino acids, and 984 nucleotide sequences encoding 327 amino acids, respectively, (Figure 11). Sequence of the full length luciferase gene was generated by nucleotide sequencer machine (First Base, Malaysia). The luciferase gene was represented by alpha subunit luciferase (Lux A) and the beta subunit luciferase (Lux B). Figure 11 indicates the positions of lux A and lux B genes in the luciferase nucleotide sequence. The sequence of the luciferase gene has been deposited in GenBank (*Accession no. KF926858*).

### 3.4. Near Real Time Field Trial

**Figure 12:**
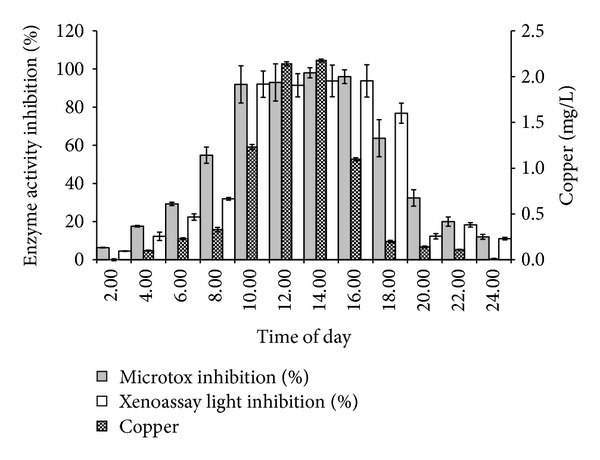
Inhibition of bioluminescence activity of Microtox and Xenoassay Light assays and copper concentration in water samples. Data is mean standard error of the mean (*n* = 3).

A time-of-the-day profile of the copper concentration in water samples of an industrial drain in the Juru Industrial Park flown into the Juru River Basin showed similar temporal inhibition between the candidate bacterial and the Microtox assays. Close correlation with inhibition of bioluminescence activity in both assays was found to copper concentrations in the water samples with temporal inhibition to both bacterial assays and elevated copper concentration occurred approximately between 10.00 and 18.00 hours (Figure 12). Heavy metal analysis of the water samples showed that the presence of the heavy metal copper with the highest concentration measured at the same spot was 2.17 mg/L occurring at 14.00 hour or 2.00 pm. The amount of other heavy metals such as mercury, silver, cadmium, and lead was negligible (data not shown).

## 4. Discussion

A luminescent bacterial strain was successfully isolated from the mackerel,* Rastrelliger kanagurta*. To the best of our knowledge this is the first report on the isolation of bioluminescent bacterium from this species. Several other bioluminescent bacteria for the purpose of bioassay have been reported such as* V. fischeri *[32, 33],* V. harveyi *[34],* V. logei *[35], and* V. fischeri *strain DSM 744 [36]. They were all isolated from marine environment with the exception of* Photorhabdus luminescens*, which was isolated from terrestrial environment [37]. Friedrich and Greenberg [38] reported that growth on carbon source other than glycerol such as glucose elicited catabolite repression of luciferase activity, hence the lag phase observed. Based on this study, glycerol was selected as the main carbon source. High luminescence activities have been observed at 3 g/L glycerol in both* P*.* phosphoreum* strain NRRL-B 1117 [29] and* P*.* phosphoreum* strain ATCC 11040 [39]. Other bioluminescent bacteria, such as* P. phosphoreum *KCTC 2852, were cultivated in luminescence media which contain 10 g/L of peptone [40] and* V. fischeri *was grown in seawater media containing 5 g/L bacto-tryptone [41]. Sodium ion supports the growth of this marine bacterium because it helps to maintain osmotic pressure. The changes of osmotic pressure will result in cell lysis [42]. In comparison,* Photobacterium* sp. strain LuB-1 has shown good sensitivity in a wide range of salt concentrations from 0.2 to 5% (w/v) [9]. A study by Cook et al. [43] shows that salinity affected greatly bioluminescence emitted by* V. fischeri* and* P. pseudomonas*.

In comparison, stable luminescence activities of* P. phosphoreum* strain KM MGU 331 were observed within the pH range of 7.0 to 9.0 [44]. Most bioluminescent bacteria require pH values close to neutral for growth and high luminescence activities [45]. For example,* P*.* phosphoreum* strain KCTC 2852 grows at pH 7.0 [40] and the optimum pH value for both growth and luminescence production of* V. fischeri *is at pH 7.5 [46].

The size of the new luciferase protein (translated) from* Photobacterium* strain MIE was compared with the previous well-studied luciferase protein from* Vibrio harveyi *(GenBank Accession no.* DQ436496.1*) to see any differences based on protein sequence. Although, many successfully isolated luciferases were reported, only the luciferases protein from* V. harveyi* was successfully crystallized so far [47]. Based on Blastp alignment, lux AB from* Photobacterium* strain MIE only showed 61% similarity to lux A and 48% similarity to lux B proteins from* V. harveyi *(result not shown). Since similarity between both luciferases was moderate, it is very interesting to know more about its protein structure and how the newly isolated luciferase could show broad optimal luminescence activity that includes room temperature (25°C–30°C).

Luminescence at these relatively high temperatures is an advantage especially for near real time biomonitoring system in a tropical country like Malaysia where the temperature is within the range of the bacterial optimal luminescent temperature range. Most of the other commonly studied bioluminescent bacteria have lower temperature for efficient bioassays than strain MIE. For example, Kim et al. [48] reported an optimum temperature of 18°C for* P. phosphoreum* strain KCTC 2852 and* P. fischeri* has an optimum temperature of 15°C [49]. The optimum temperature for both* V. fischeri* and* P. phosphoreum* strain A-13 is 15°C [9, 50, 51]. In order to maintain stability of the luminescence produced by strain MIE, the bacterial cultures should be harvested between 8 and 24 hours of incubation. This result conforms to the study done by Girotti et al. [14] that shows the relationship between luminescence peak of* V. logei *and its growth curve.

The results demonstrated near real time capability of the proposed system. The concentrations of copper exceeded the maximum permissible limit (MPL) outlined by the DOE Malaysia at 0.02 mg/L for class II (DOE 2011) [1]. The errors of the inhibition measurement (coefficient of variation) were less than 10% (*n* = 5) suggesting good reproducibility. Strain MIE exhibited high tolerance when subjected to a wide range of environmental factors. This is essential as the application of the bioluminescent assay in various samples requires a luminescent strain with better sensitivity and stability in diverse assay conditions. This work shows that strain MIE is also capable of producing significant luminescence at slightly acidic environment. The bioluminescence process, as well as growth, can be suppressed at certain pHs due to the inhibition of enzymes involved in the bacterial metabolic reactions and the instability of the transport protein [52]. In this case, a wide range of pHs for luminescence is a strong advantage for water monitoring, especially in heavy metal contaminated sites, as deviation in sample pHs can be highly tolerated by this strain.

A field trial performed in an industrial site that channels its waters into agricultural areas in Penang showed the practicality of this strain for water quality biomonitoring in the tropical region. Simple methodology, rapid assay time, and reproducible data are major advantages in any field assessment. The fluctuation and variation in water temperature can change the metabolic response of the strain used in the assay and greatly affect the luminescence production [13]. Other bioluminescent bacteria such as* V. fischeri *and* P. phosphoreum* have optimum temperatures at 15°C [53]. These bacteria are not suitable for real time monitoring studies in tropical areas which can generally have temperature of more than 15°C. Any assays utilizing these bacteria under tropical conditions can be costly due to the requirement of expensive refrigeration or incubation. Our works show that this strain performed successfully at ambient temperature. Furthermore, the toxicity assay on copper-contaminated water was also demonstrated to be fast and precise. Bioassay using this strain has the potential to be cheaper, faster, and more stable than other classical toxicity bioassays. This proposed luminescence system should be able to address problems and issues related to a large scale biomonitoring of toxicants in rivers basins that fed into agricultural areas [54, 55].

Future works include the determination of half maximal inhibitory concentration (IC_50_) of selected heavy metals, pesticides, and other xenobiotics to the bioluminescence system and a large scale field trial work assessment of the proposed system in comparison with ToxAlert, a near real time system, and the classical Microtox, which are all based on* V. fischeri*.
